# Depression Screening as a Part of the Employees' Annual Checkup Using a Two-Step Approach at King Khalid University Hospital, Riyadh, Saudi Arabia

**DOI:** 10.7759/cureus.66092

**Published:** 2024-08-03

**Authors:** Rakan Alsalem, Saleh Albahlei

**Affiliations:** 1 Department of Family and Community Medicine, College of Medicine, King Saud University, King Saud University Medical City, Riyadh, SAU

**Keywords:** healthcare worker, screening, prevalence, patient health questionnaire (phq-9), depression

## Abstract

Introduction

Health professionals experience high levels of work-related stress; hence, the study of depression among healthcare workers (HCWs) is essential to improve patient care, prevent burnout, and retain a skilled workforce as well as reduce stigma, enhance workplace productivity, and promote overall well-being. This study aimed to estimate the prevalence of depression and associated factors among HCWs at King Khalid University Hospital, Riyadh, Saudi Arabia.

Methods

We conducted a cross-sectional study among HCWs at King Khalid University Hospital. All healthcare workers required to renew their contract annually were given a Patient Health Questionnaire 2 (PHQ2) tool for screening for depression; if their score was three or more, a Patient Health Questionnaire 9 (PHQ9) tool was given along with additional questions including demographic, profession-related factors, and stressor presence in different life dimensions.

Results

In total, 69 HCWs filled out the screening survey (PHQ9). Most were females (n=57; 82.6%), with 36 (52.2%) aged 35 years or below. Five (7.2%) HCWs reported a family history of mental illness. The prevalence of major depressive disorders among HCWs was 29 (42%).

Conclusions

Younger HCWs who smoked and had no children were more susceptible to depression. Annual psychological screenings for HCWs could be beneficial for monitoring staff vulnerable to mental health disorders. We need a future multicenter study approach to confirm the prevalence of major depression in our region.

## Introduction

Depression is a common mental illness characterized by low mood, decreased interest or pleasure, reduced energy, feelings of guilt or low self-worth, disrupted sleep or appetite patterns, and difficulties with concentration. According to the World Health Organization (WHO), approximately 280 million people worldwide are affected by depression. Furthermore, depression is the leading cause of disability worldwide; it affects both genders and contributes to 4.4% of all disability cases [1]. Healthcare workers (HCWs) face heightened susceptibility to anxiety and depression disorders due to their exposure to significant occupational stressors [2-4]. A systemic review conducted in 2021 across 13 countries revealed a pooled depression prevalence of 33.03% among HCWs in the Eastern Mediterranean Region (EMR) between 2005 and 2020 [5]. Attention to depression among HCWs is crucial, given its significant implications for public health. This importance arises not only from the nature of their profession and its direct impact on others' health but also from HCWs' vulnerability to specific hazards such as medication errors, malpractice, substance misuse, and suicide [3].

Several studies have investigated the prevalence of depression and anxiety among HCWs in Saudi Arabia [2,5-7]. The Ministry of Health (MOH) conducted studies to estimate the prevalence of depression and anxiety disorders among HCWs, exploring potential predisposing factors and related risks. One such study revealed that the most common type of work-related stress among HCWs was due to job pressure, followed by poor rapport with managers [2]. Other frequent stressors included long working hours, lack of appreciation, and inadequate breaks. In another study conducted at the National Guard Hospital, HCWs at King Abdul Aziz Medical City (KAMC) were surveyed using a self-administered questionnaire [6]. The findings indicated that 11.4% of the study population exhibited symptoms of depression, with 6.7% diagnosed with major depressive disorder. Similarly, in Tabuk City, a group of researchers investigated the frequency and causes associated with depression among HCWs [5]. They found that the prevalence of depression was 43.9%, with 0.8% of cases being severe. HCWs who lost a family member in the last six months faced an increased likelihood of developing depression. Furthermore, compared to HCWs with less than five years of experience, those with more experience were at a significantly lower risk of developing depression [7].

This study is the first to investigate depression among HCWs at King Khalid University Hospital, Riyadh, Saudi Arabia. It aims to estimate the prevalence and assess the levels of depression. Additionally, it explores the associated determinants, which could have significant public health implications for higher authorities.

## Materials and methods

This cross-sectional study was conducted among HCWs at King Khalid University Hospital in Riyadh, Saudi Arabia. The study was approved by the King Saud University Institutional Review Board (Approval number: 23/0885/IRB, dated 05/12/2023) and adhered to internationally recognized codes and standards of research ethics.

All HCWs required to renew their contracts annually were given the Patient Health Questionnaire 2 (PHQ-2) tool for depression screening as part of a screening program implemented in 2021. Responses from 2021 to 2023 were included. If a worker's PHQ-2 score was three or more, they were given the Patient Health Questionnaire 9 (PHQ-9) tool with additional questions covering demographic and professional factors and stressors in different life dimensions. A total of 3,630 participants were screened with the PHQ-2. Patients who scored 3 or more on the PHQ-2 were contacted, and a follow-up survey including the PHQ-9 and additional data was sent to them. Out of 110 people with a positive PHQ-2, 69 completed the PHQ-9, resulting in a response rate of 75.9%.

Questionnaire criteria

Depression was assessed using the PHQ-9 (see Appendices) developed by Kroenke et al. [8]. This is a nine-item questionnaire with four-point Likert scale categories ranging from “not at all” coded with 0 to “nearly every day” coded with 3. The severity of depression is classified as minimal (score <5), mild (score 5-9), moderate (score 10-14), moderately severe (score 15-19), and severe (score 20-27). For the purposes of this study, the severity levels were reclassified into two categories: major depressive disorder (score >10) and no depressive disorder (score ≤10).

Statistical analysis

The data were analyzed using IBM SPSS Statistics for Windows, Version 26.0 (Released 2019; IBM Corp., Armonk, New York, United States). Categorical variables were shown as numbers and percentages. Continuous variables were calculated and given as mean and standard deviation. The relationship between major depressive disorder and the socio-demographic characteristics of participants has been conducted using the Chi-square test. Significant results were then tested in a multivariate regression analysis to determine the significant independent risk factor of major depressive disorder. Statistical significance was set to p<0.05.

## Results

Starting in 2021, all HCWs were screened annually for depression, among other comorbidities such as diabetes, hypertension, obesity, and smoking, as part of their annual contract renewal process. A total of 3,633 workers were screened using the PHQ-2, and 69 workers who scored three or more were contacted and sent a follow-up questionnaire that included the PHQ-9 screening tool, representing 3.03% of the initial screening population.

As shown in Table 1, 36 (52.2%) HCWs were aged 35 years or younger, with a female majority of 57 (82.6%). Regarding marital status, 34 (49.3%) HCWs were married, and six (8.7%) had more than two children. Approximately 41 (59.4%) were nurses, and 44 (63.8%) study participants had 10 years or less of working experience. The proportion of HCWs who had on-call duties was 27 (39.1%). Daily working hours were mostly nine hours (n=31; 44.9%), and 32 (46.4%) worked regular shifts. The majority of HCWs received peer support (n=45; 65.2%) and had bachelor’s degrees 55 (79.7%). In terms of monthly income, 44 (63.8%) earned less than 10,000 SAR monthly. Only 10 (14.5%) reported engaging in physical activity more than three times per week. Five (7.2%) had a family history of mental disorders, with depression being the most common (n=2; 40%). The prevalence of smoking among HCWs was 15 (21.7%), and two (2.9%) had a previous history of substance abuse.

**Table 1:** Sociodemographic characteristics of healthcare workers (N=69)

Study variables	Frequency (Percentage)
Age group	
≤35 years	36 (52.2%)
>35 years	33 (47.8%)
Gender	
Male	12 (17.4%)
Female	57 (82.6%)
Marital status	
Single	31 (44.9%)
Married	34 (49.3%)
Divorced or widowed	04 (05.8%)
Have Children	
No	39 (56.5%)
Yes	3012 (43.5%)
Working Position	
Doctor	18 (26.09%)
Nurse	41 (59.42%)
Lab Technician	10 (14.49%)
Years of Experience	
≤10 years	44 (63.8%)
>10 years	25 (36.2%)
On-call Duties	
No	42 (60.9%)
Yes	27 (39.1%)
Daily Working Hours	
8-9 Hours	40 (57.97%)
>9 Hours	29 (42.03%)
Working in Shifts (n= 54)	
Regular	32 (46.4%)
Mixed	22 (31.9%)
No	15 (21.7%)
Peer Support (e.g., close friend or family member)	
No	24 (34.8%)
Yes	45 (65.2%)
Level of Education	
High school	04 (05.8%)
Bachelor degree	55 (79.7%)
Higher education	10 (14.5%
Monthly income (SAR)	
<10,000	44 (63.8%)
>10,000	25 (36.2%)
Physical activity weekly (e.g., Gym, walks, etc.)	
No	24 (34.8%)
Yes	45 (65.2%)
Family history of mental diseases	
No	64 (92.8%)
Yes	05 (7.2%)
Specify (n=5)	
Depression	02 (40.0%)
Anxiety	01 (20.0%)
Autism	01 (20.0%)
Bipolar	01 (20.0%)
Smoking	
No	54 (78.3%)
Yes	15 (21.7%)
History of Substance Abuse	
No	67 (97.1%)
Yes	02 (02.9%)

**Figure 1:**
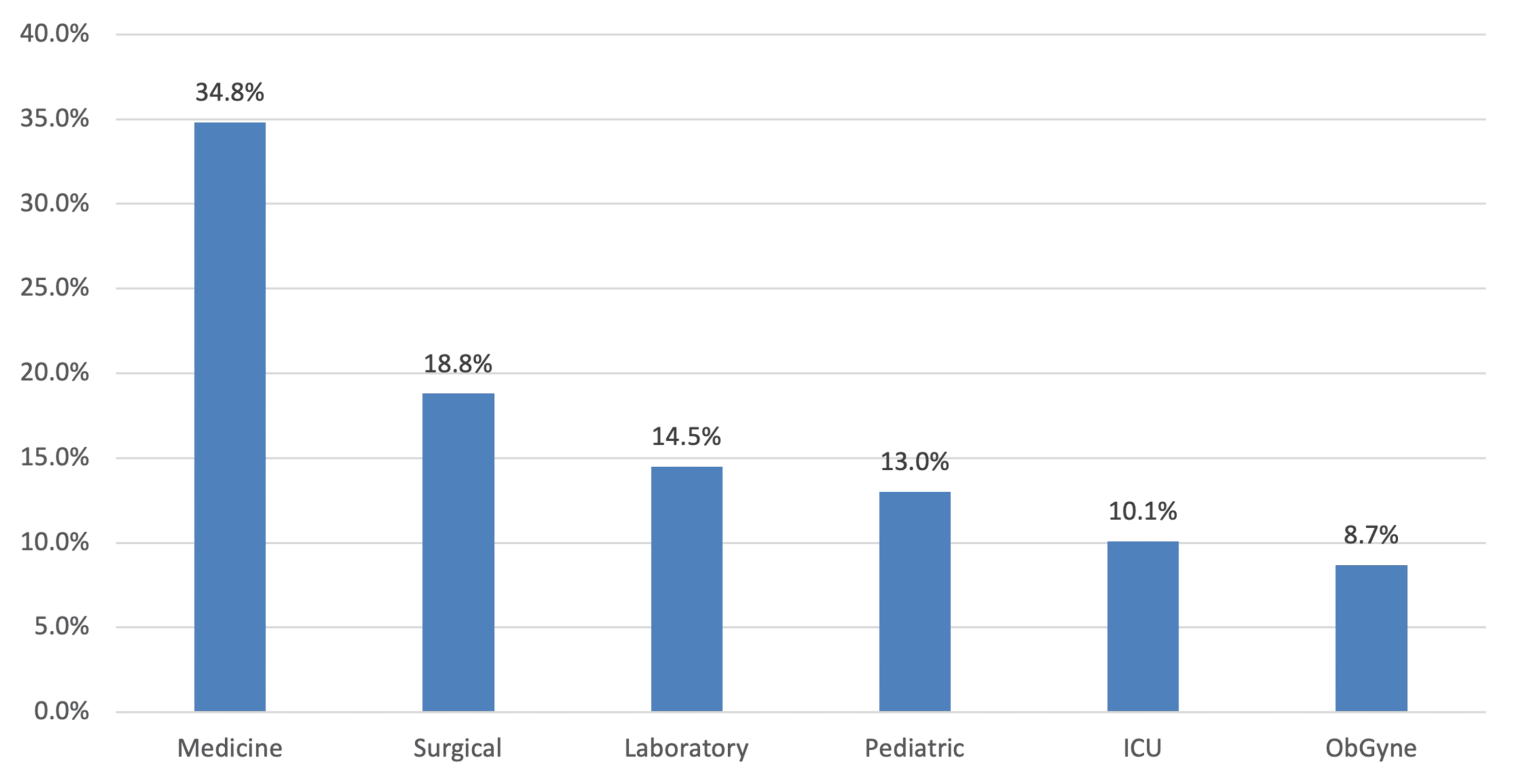
Working department

As displayed in Figure 1, the most common working department among HCWs was medicine, accounting for 24 (34.8%) of the total. This was followed by the surgery department, with 13 (18.8%), and the laboratory department, with 10 (14.5%).

According to Table 2, the study found that the overall mean depression score among the participants was 8.48 (± 6.62 SD). According to the criteria used, 29 (42%) of the participants were classified as having major depressive disorders, while the remaining 40 (58%) were considered normal. In terms of depression severity, 15 (21.7%) were categorized as having moderate depression, 10 (14.5%) as moderately severe, and only four (5.8%) as severe.

**Table 2:** Prevalence of major depressive disorder using PHQ-9. PHQ-9: Patient Health Questionnaire 9

Variables	Values
Depression score, mean ± SD	8.48 ± 6.62
Major depressive disorder	Frequency (Percentage)
Yes (score ≥10)	29 (42.0%)
No (score <10)	40 (58.0%)
Depression Severity	
Minimal (score 1 – 4)	24 (34.8%)
Mild (score 5 – 9)	16 (23.2%)
Moderate (score 10 – 14)	15 (21.7%)
Moderately severe (score 15 – 19)	10 (14.5%)
Severe (score 20 – 27)	04 (05.8%)

As shown in Table 3, when examining the relationship between major depression and the sociodemographic characteristics of HCWs, we found that HCWs who exhibited symptoms of major depression were more likely to be younger (p=0.001), have children (p=0.001), possess fewer years of experience (p=0.022), and were smokers (p=0.029).

**Table 3:** Relationship between major depressive disorder and sociodemographic characteristics of HCWs (N=69) § P-value has been calculated using Chi-square test. ** Significant at p<0.05 level. HCW: healthcare workers; SAR: Saudi Arabian Riyal

Factor	Major depressive disorder		P-value ^§^
	Yes (n=29), n (%)	No (n=40), n (%)	
Age Group			
≤35 years	22 (75.9%)	14 (35.0%)	0.001 **
>35 years	7 (24.1%)	26 (65.0%)	
Gender			
Male	05 (17.2%)	07 (17.5%)	0.978
Female	24 (82.8%)	33 (82.5%)	
Marital Status			
Unmarried	18 (62.1%)	17 (42.5%)	0.109
Married	11 (37.9%)	23 (57.5%)	
Have Children			
No	23 (79.3%)	16 (40.0%)	0.001 **
Yes	06 (20.7%)	24 (60.0%)	
Working Position			
Non-nurse	15 (51.7%)	13 (32.5%)	0.108
Nurse	14 (48.3%)	27 (67.5%)	
Years of Experience			
≤10 years	23 (79.3%)	21 (52.5%)	0.022 **
>10 years	06 (20.7%)	19 (47.5%)	
On-call Duties			
No	18 (62.1%)	24 (60.0%)	0.862
Yes	11 (37.9%)	16 (40.0%)	
Daily Working Hours			
8-9 hours	16 (55.2%)	24 (60.0%)	0.688
>9 hours	13 (44.8%)	16 (40.0%)	
Working Shifts			
Regular	12 (41.4%)	20 (50.0%)	0.648
Mixed	11 (37.9%)	11 (27.5%)	
No	06 (20.7%)	09 (22.5%)	
Peer Support			
No	10 (34.5%)	14 (35.0%)	0.964
Yes	19 (65.5%)	26 (65.0%)	
Monthly Income (SAR)			
<10,000	18 (62.1%)	26 (65.0%)	0.803
≥10,000	11 (37.9%)	14 (35.0%)	
Physical Activity Weekly			
No	13 (44.8%)	11 (27.5%)	0.136
Yes	16 (55.2%)	29 (72.5%)	
Family History of Mental Diseases			
No	25 (86.2%)	39 (97.5%)	0.074
Yes	04 (13.8%)	01 (02.5%)	
Smoking			
No	19 (65.5%)	35 (87.5%)	0.029 **
Yes	10 (34.5%)	05 (12.5%)	

Table 4 showed significant results; a multivariate regression analysis was subsequently performed to determine the significant independent risk factors for major depressive disorders. Based on the results, it was observed that while age (adjusted OR (aOR)=0.268; 95%CI=0.057 - 1.271; p=0.097) and having children (aOR=0.304; 95% CI=0.077 - 1.194; p=0.088) were at lower risk of having a major depressive disorder, and years of experience (aOR=1.535; 95%; CI=0.277 - 8.503; p=0.624; p=0.624), as well as smoking (AOR=1.746; 95% CI=0.466 - 6.546; p=0.409) were at higher risk of major depression; however, the overall results did not reach statistical significance after adjustment to a regression model.

**Table 4:** Multivariate regression analysis to determine the significant independent predictors of major depressive disorders (N=69) **  Significant at p<0.05 level.

Factor	Adjusted OR	95% CI	P-value
Age Group			
≤35 years	Ref		
>35 years	0.268	0.057 – 1.271	0.097
Have children			
No	Ref		
Yes	0.304	0.077 – 1.194	0.088
Years of Experience			
≤10 years	Ref		
>10 years	1.535	0.277 – 8.503	0.624
Smoking			
No	Ref		
Yes	1.746	0.466 – 6.546	0.409

## Discussion

This study investigated the prevalence and influential factors of major depressive disorders among HCWs at King Khalid University Hospital. Using the PHQ-2 assessment tool, only 3.03% of the population had a positive score (score of 3 or more; n=69). Upon further assessment of these workers using the PHQ-9 tool, 42% were considered to have a major depressive disorder. Several studies reported that depression among healthcare professionals was not uncommon [2-5,7,9-12]. One study conducted in Riyadh found the rate of depression among HCWs at 6.7%, with only 11.4% exhibiting signs of depression [13]. In contrast, a study conducted in Iraq revealed the highest depression rate among HCWs at 85% [9]. Regional differences may significantly contribute to the variation in depression rates. Some studies propose that elevated prevalence rates might be associated with income levels and the organizational framework of the workplace [11], while others associate it with traumatic events encountered in the workplace [9].

The data from this study suggest that younger HCWs aged under 35 and those without children were more prone to experiencing depression compared to older individuals (≥35 years) or those with children (p<0.05). This finding aligns with the research by Alwhaibi et al. [2]. Factors such as age, social, and financial status showed a positive correlation with overall burnout scores, and all burnout subscales were higher among depressed HCWs. However, despite literature indicating that being female and unmarried increased the risk of depression [3,8,11,12], our results did not show a significant relationship between gender, marital status, and depression (p>0.05). HCWs who have fewer years of experience may be more susceptible to developing major depressive symptoms. This is consistent with findings from AlFahhad's study in 2018, which showed a significantly higher prevalence of depression among HCWs in their first year of work [13]. Similarly, Almarhapi and Khalil found that increasing years of experience were associated with a decreased risk of depression [7]. Although smoking was observed to increase the risk of major depression, this finding did not reach statistical significance after adjustment in a regression model (p=0.409). Similarly, factors such as working shifts, on-call duties, and a family history of mental disease did not influence the development of major depression (p>0.05). This contrasts with some studies in the literature that recognized a family history of mental illness and substance abuse as significant predictors of exhibiting depressive symptoms [12]. Furthermore, studies conducted in Jeddah [3] and Qassim [6] emphasized that work-related stressors significantly affect the mental health of healthcare workers.

Coping mechanisms are crucial for preventing the progression of mental illnesses such as depression. Liang et al. reported that no previous history of psychological intervention and perceived good ratings of health status were identified as protective factors for depression [14]. Additionally, resilience was found to be inversely proportional to co-occurring anxiety and depression. In our study, the potential protective effects of peer support and regular exercise did not substantially align with the development of depression. Therefore, further investigations are warranted to determine their impact.

The current study should be considered within its limitations. First, our analysis included a limited sample size (n=69) and has convenient sampling method. While this number may reflect the true prevalence of depression in our population, it could also be significantly underestimated as workers may fear that positive responses could affect their contract renewal or position. A larger sample size would likely yield more accurate results, providing a more comprehensive picture of the prevalence and factors influencing depression. Second, the gender distribution was unequal, limiting the generalizability of gender-based comparisons. Third, the questionnaire did not include structured coping mechanisms, which should be addressed in future research. Lastly, as a cross-sectional survey, this study is prone to bias and cannot establish causality.

## Conclusions

Our study demonstrated that depressive symptoms among HCWs were not uncommon. Younger HCWs, smokers, and those without children were more susceptible to depression. Annual psychological screening for HCWs could be beneficial in monitoring staff vulnerable to mental health disorders. Furthermore, programs to enhance resilience and coping mechanisms are crucial for improving the psychological well-being of HCWs.

## Appendices

**Table 5:** The Patient Health Questionnaire (PHQ-9) Source: Kroenke et al., 2001 [8]

Name ______________________ Date _________				
Over the *last 2 weeks*, how often have you been bothered by any of the following problems?	Not at all	Several days	More than half the days	Nearly every day
1. Little interest or pleasure in doing things	0	1	2	3
2. Feeling down, depressed, or hopeless	0	1	2	3
3. Trouble falling or staying asleep, or sleeping too much	0	1	2	3
4. Feeling tired or having little energy	0	1	2	3
5. Poor appetite or overeating	0	1	2	3
6. Feeling bad about yourself—or that you are a failure or have let yourself or your family down	0	1	2	3
7. Trouble concentrating on things, such as reading the newspaper or watching television	0	1	2	3
8. Moving or speaking so slowly that other people could have noticed? Or the opposite—being so fidgety or restless that you have been moving around a lot more than usual	0	1	2	3
9. Thoughts that you would be better off dead or of hurting yourself in some way	0	1	2	3
(For office coding: Total Score ____ = ____ + ____ + ____)				
